# Computational Intelligence-Based Stuttering Detection: A Systematic Review

**DOI:** 10.3390/diagnostics13233537

**Published:** 2023-11-27

**Authors:** Raghad Alnashwan, Noura Alhakbani, Abeer Al-Nafjan, Abdulaziz Almudhi, Waleed Al-Nuwaiser

**Affiliations:** 1 Information Technology Department, College of Computer and Information Sciences, King Saud University, Riyadh 11543, Saudi Arabia; 444203361@student.ksu.edu.sa (R.A.); nhakbani@ksu.edu.sa (N.A.); 2 Computer Science Department, College of Computer and Information Sciences, Imam Mohammad Ibn Saud Islamic University (IMSIU), Riyadh 11432, Saudi Arabia; wmalnuwaiser@imamu.edu.sa; 3 Department of Medical Rehabilitation Sciences, College of Applied Medical Sciences, King Khalid University, Abha 62529, Saudi Arabia; almudhi@kku.edu.sa

**Keywords:** stuttering detection, systematic review, rehabilitation, machine learning

## Abstract

Stuttering is a widespread speech disorder affecting people globally, and it impacts effective communication and quality of life. Recent advancements in artificial intelligence (AI) and computational intelligence have introduced new possibilities for augmenting stuttering detection and treatment procedures. In this systematic review, the latest AI advancements and computational intelligence techniques in the context of stuttering are explored. By examining the existing literature, we investigated the application of AI in accurately determining and classifying stuttering manifestations. Furthermore, we explored how computational intelligence can contribute to developing innovative assessment tools and intervention strategies for persons who stutter (PWS). We reviewed and analyzed 14 refereed journal articles that were indexed on the *Web of Science* from 2019 onward. The potential of AI and computational intelligence in revolutionizing stuttering assessment and treatment, which can enable personalized and effective approaches, is also highlighted in this review. By elucidating these advancements, we aim to encourage further research and development in this crucial area, enhancing in due course the lives of PWS.

## 1. Introduction

Stuttering, a prevalent speech disorder that affects millions all over the globe [1], lacks comprehensive research in terms of accurately determining and categorizing its manifestations. Even though speech is a fundamental medium to convey ideas and emotions, not all individuals can flawlessly verbally communicate. The efficacy of speech is dependent on its fluency, which denotes the natural flow between phonemes that constitute a message [2]. Dysfluencies, which include stuttering, disrupt this flow and represent a complexity that impacts over 80 million people worldwide, that is, approximately 1% of the world’s population [3].

Stuttering is characterized by the repetition of sounds, syllables, or words; the prolongation of sounds; and the interruption of speech through blocks. PWS often have a clear understanding of their intended speech but struggle with its fluid expression. These disruptions in speech can manifest with accompanying struggle behaviors, including secondary behaviors such as rapid eye blinks and quivering lip movements. The impacts of stuttering extend beyond its surface manifestations; it impairs effective communication, thus affecting interpersonal relationships and the overall quality of life of people suffering from it [4]. People with varying degrees of stuttering severity may find difficulties in both social interactions and professional settings, with heightened severity correlated with potential emotional struggles [5].

The traditional approach to evaluating stuttering is to manually tally the instances of different stuttering types and express them as a ratio that is relative to the total words in a speech segment. Nevertheless, owing to its time-intensive and subjective nature, this method is not without limitations that lead to inconsistencies and potential errors when different evaluators are involved [2]. Manually detecting stuttering exhibits several challenges. First, distinguishing stuttering from other speech disfluencies can be difficult, considering that subtle instances may resemble hesitations or pauses. Furthermore, consistent detection of stuttering becomes a complex task because the severity and frequency of stuttering can vary widely among people and across different contexts. Moreover, factors such as the speaker’s age, gender, and language as well as the speaking task and the context in which the speech is produced can further complicate the identification of stuttering [6].

Considering the increasing need for improved detection and management of stuttering, there is a noticeable trend in adopting innovative technologies, particularly artificial intelligence (AI) [7]. The application of AI in identifying and classifying stuttering indicates an essential development in the study of speech-related issues. AI has a special ability to understand complex speech patterns that might not be easy for humans to notice, and this capability can help in the early detection of stuttering. This potential has sparked a wave of novel research efforts, each influenced by the prospect of revolutionizing the understanding and treatment of stuttering. The way AI and stuttering research work together can modify how speech assessment and management are carried out and enhance the quality of life of PWS. This exciting progress demonstrates that AI can greatly assist in managing stuttering and can even alter how speech therapy is performed, which makes it more personalized and effective.

This study highlights a perspective on the utilization of AI technologies for determining and classifying stuttering. While AI holds promise for the assessment of stuttering, this area has received limited attention, likely due to the complexity of the disorder, the need for extensive and diverse datasets, and the challenges of developing robust and accurate AI models. This study analyzes the existing research to extract recent efforts and methods in the field. The primary objective is to categorize and summarize the relevant literature concerning to stuttering identification, offering insights and organizing these articles for future research focused on the use of AI in stuttering identification. This approach aims to facilitate advancements in the field by highlighting the recent developments and methodologies employed in automated stuttering identification.

In the field of ASD research, it is essential to acknowledge the prior systematic reviews that have explored machine learning approaches for stuttering identification. Two notable systematic reviews have been published in recent years, namely Sheikh et al. (2022) [8] and Barrett et al. (2022) [9], who conducted a comprehensive review that encompassed various aspects of stuttering identification, including stuttered speech characteristics, datasets, and automatic stuttering-identification techniques. On the other hand, Barrett et al. focused specifically on machine learning techniques for detecting developmental stuttering. Their systematic review concentrated on studies utilizing supervised learning models trained on speech data from individuals who stutter. They emphasized the importance of accuracy reporting, sample sizes, and specific inclusion criteria in their analysis.

This study aims to answer the following questions: (i) What are the recent advancements in AI and computational intelligence for stuttering detection and treatment, and how can they contribute to improving assessment and intervention strategies? (ii) What are the challenges and future directions in computational intelligence-based stuttering detection, and how can they be addressed to enhance accuracy and effectiveness?

Our review aims to differentiate itself by providing a distinct contribution to the field. We focus on the latest developments in AI and computational intelligence within the timeframe of 2019–2023, specifically addressing the challenges and advancements in stuttering detection. Our review offers a comprehensive analysis of the datasets used, the specific types of stuttering investigated, the techniques employed for feature extraction, and the choice of classifiers. Furthermore, we aim to highlight potential pathways for improving accuracy and effectiveness in computational intelligence-based stuttering detection.

The rest of this paper is structured as follows: Section 2 explains the method utilized for this organized review. Section 3 covers the results and discussion. Section 4 provides insights for future research. Finally, Section 5 presents the conclusions.

## 2. Research Methodology

Research articles that are focused on automating the identification of stuttering are spread throughout different conference proceedings and journals, encompassing distinct areas for enhancement. These areas include refining methods for improving stuttering-identification accuracy, assessing various forms of stuttering, refining severity evaluation, and enhancing accessible datasets. This section describes the approach employed to locate relevant articles, along with the criteria for article selection and the procedures for filtering. Despite being a relatively novel and emerging field, research into stuttering detection using AI has received attention from researchers from various fields, eventually becoming a significant area of academic investigation. The outcomes of studies in this domain have been published in scholarly journals and conferences and indexed in the *Web of Science* (*WoS*) database [10].

**Figure 1:**
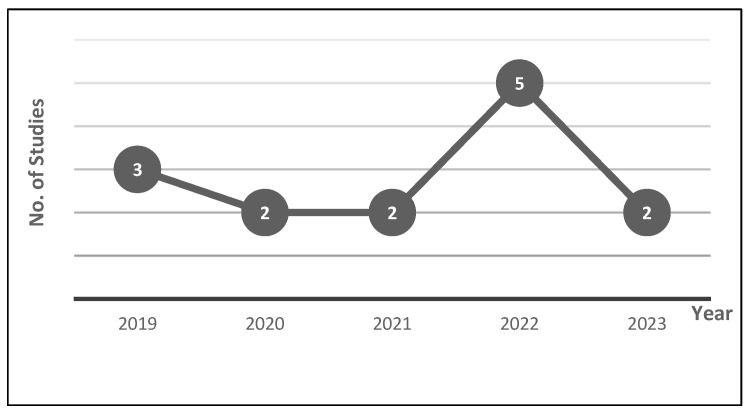
Number of published articles per year.

The *Web of Science* (*WoS*) database is widely recognized for its comprehensive coverage of academic literature and its commitment to delivering high-quality research. The *WoS Core Collection* database provides us with robust access to prominent citation databases, including *Science Direct* (Elsevier), *IEEE/IEE Library*, *ACM Digital Library*, *Springer Link Online Libraries*, and *Taylor & Francis*. Utilizing the WoS for research provides numerous advantages owing to its comprehensive coverage of scholarly literature. This robust platform provides access to an extensive range of high-quality journals, conference proceedings, and research articles from different disciplines. The platform’s precise indexing and citation tracking help researchers determine key studies and trends. Moreover, its rigorous evaluation and inclusion of reputable sources enhance the reliability and credibility of accessed materials, elevating the overall quality of research efforts. Articles and review articles published between 2019 and 2023 were precisely searched within the *WoS Core Collection* database (Figure 1).

**Figure 2:**
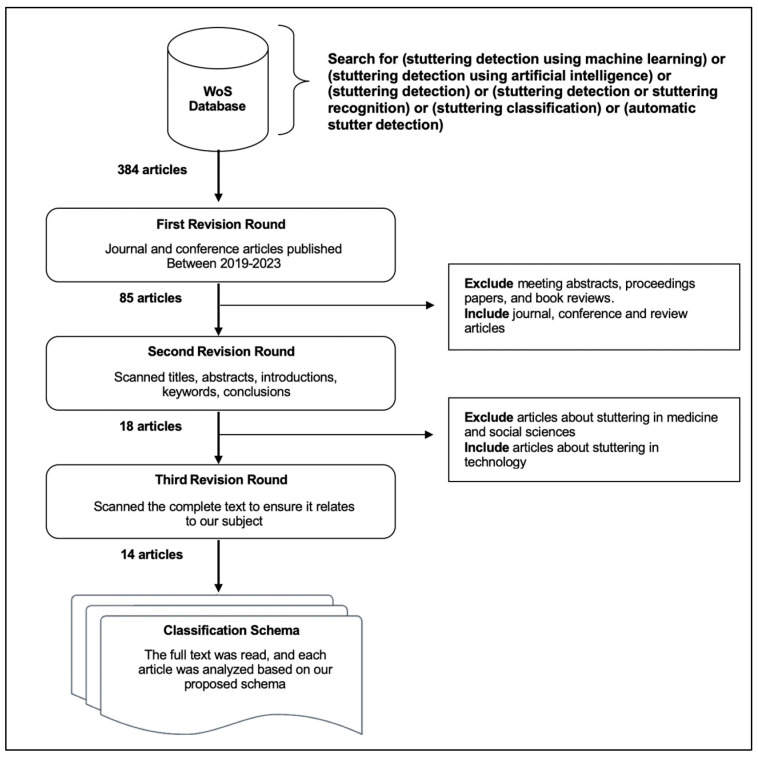
Article filtering process.

By using the search field, to guarantee the inclusion of all studies pertinent to the identification of stuttering via several AI technologies, we entered the following terms: (stuttering detection using machine learning) or (stuttering detection with the use of AI) or (stuttering detection) or (stuttering detection or stuttering recognition) or (stuttering classification) or (automatic stutter detection). Figure 2 illustrates the search process.

Initially, the search yielded a total of 384 research papers. From this pool, we selected journal and conference articles for inclusion and excluded meeting abstracts, proceedings papers, and book reviews. This selection also focused on articles published between 2019 and 2023. Following this initial stage, we finally obtained a refined collection of 85 search results. Afterward, a secondary screening procedure was performed, where we conducted an in-depth manual assessment to identify whether each article was relevant to the subject matter. Consequently, any articles that were considered unrelated were excluded from consideration.

In the second phase of revision, we reviewed the titles, abstracts, introductions, keywords, and conclusions of the articles. Then, we refined the collection by specifically choosing papers that centered around the application of machine learning and AI in stuttering detection. This yielded a set of 18 articles. In the third phase of revision, we meticulously examined the complete texts of the remaining 18 articles. After careful consideration, four articles were found to be unrelated to our designated theme, as they focused on the medical field. Consequently, these articles were excluded from our analysis, resulting in a final selection of 14 articles.

These 14 articles served as the basis for a further in-depth analysis and comparison of important aspects in automatic stuttering-detection (ASD) research. Our analysis was conducted based on specific criteria, taking into account the need for a comprehensive evaluation. These criteria included the dataset utilized in each study, the specific type of stuttering investigated (such as prolongation, block, or repetition), the techniques employed for feature extraction, the choice of classifier used, and the achieved performance accuracy.

By considering these five dimensions—dataset, classified stuttering type, feature-extraction approach, classifier selection, and performance evaluation—we aim to provide a structured and comprehensive overview of the diverse studies within the field of ASD. This framework will facilitate a deeper understanding and effective comparison of the different research approaches in this domain.

## 3. Results

### 3.1. Datasets

The success of stuttering detection using deep learning and AI models crucially depends on the quality and diversity of the data applied for training these systems. This critical dependency underscores the need for encompassing datasets that capture a wide spectrum of stuttering patterns, speech variations, and linguistic contexts.

In the literature, researchers have applied various datasets, including *University College London’s Archive of Stuttered Speech* (*UCLASS*) [11], *SEP-28k* [12], *FluencyBank* [13], and *LibriStutter* [14], and have also used resources like *VoxCeleb* [15] (see Table 1). Furthermore, some researchers such as [16,17,18] have even made their own customized datasets to cater to their specific research needs. This combined endeavor emphasizes the importance of carefully curated and extensive data in advancing the field of stuttering-detection technology via deep learning and AI methods. Table 1 presents the benchmark datasets along with their respective descriptions.

In Table 1, the dataset most commonly applied is *UCLASS*, which has been utilized in studies [14,19,20,21,22,23,24], closely followed by the *SEP-28k* dataset, which is featured in various works [6,23,25,26,27]. Notably, the latter dataset has gained popularity in recent research endeavors. *FluencyBank* contributes to investigations in previous studies [6,23,24], whereas the utilization of the *LibriStutter*/*LibriSpeech* dataset is relatively less frequent, as seen in previous studies [6,25]. Conversely, the *VoxCeleb* dataset plays a more minor role, appearing only once in [25].

Furthermore, several studies [16,17,18] chose to create their own tailored datasets to address their specific research objectives. For instant, in [16], the dataset comprised 20 individuals aged between 15 and 35, all of whom had been diagnosed with stuttering by qualified speech language pathologists. This group consisted of 17 males and 3 females. They were all directed to read a specific passage, and their speech was recorded in a room at the All India Institute of Speech and Hearing (AIISH) in Mysuru using the PRAAT tool, which has a sampling rate of 44 KHz. The participants displayed characteristics of stuttering, including repeating sounds or syllables, prolongations, and blocks.

Pravin et al. [17] created a dataset that consists of recordings of natural speech from children who came from bilingual families (speaking both Tamil and English). The children included in the dataset were between the ages of 4 and 7. Furthermore, the dataset included recordings of the pronunciation of phonemes as well as mono-syllabic and multi-syllabic words in both English and Tamil.

Asci et al. [18] recruited 53 individuals with stuttering (24 females and 29 males aged 7–30) alongside 71 age- and sex-matched controls (29 females and 44 males aged 7–30). All participants were native Italian speakers, non-smokers, and had no cognitive or mood impairments, hearing loss, respiratory disorders, or other conditions that could affect their vocal cords. None were taking central nervous-system-affecting drugs at the time of the study, and demographic and anthropometric data were collected during the enrollment visit.

Some studies [14,16,17,18,19,20,21,22,26,27] used just one dataset, whereas others [6,23,24,25] opted for multiple datasets. In one particular study [6], the researchers went a step further by enhancing their data with the *MUSAN* dataset for added diversity. In terms of stuttering, implementing data augmentation can be difficult, considering that many common techniques such as time stretch and high rate of speech fundamentally alter the structure of disfluent speech samples. The proposed approach, however, uses techniques such as speech shadowing, threshold masking, and delayed auditory feedback, which closely mimic real-world conditions without significantly altering the underlying stuttering characteristics of the speech sample. This diversity in dataset choice and augmentation methods showcases the evolving nature of research approaches in this field.

### 3.2. Classified Stuttering Type

Diverse forms of stuttering have been the subject of investigation across various research studies. These encompass repetition, prolongation, block, interjection, sound repetitions, part-word repetitions, word repetitions, phrase repetitions, syllable repetition, and revision.

Table 2 illustrates each type and its definition. Among these categories, prolongation emerged as the most frequently explored, being referenced in 13 out of the 14 studies, followed by interjection, which was cited in 9 out of the 14 studies. Contrarily, part-word repetition and syllable repetition were the least discussed, having only a single mention each.

The predominant stuttering categories, namely repetition, prolongation, and block, were discussed in previous works [6,16,18,22,24]. Other variations of stuttering, which can be considered subcategories of the primary classes, were addressed in previous studies [14,19,20], including sound repetitions, word repetitions, and phrase repetitions. Interjection was highlighted in various studies [6,14,19,20,23,24,25], whereas sound repetition and word repetition were jointly explored in different studies [21,23,26,27]. Additional examinations of prolongation, block, and interjection occurred in studies [26,27]. Revisions and prolongations were linked in previous works [14,20], and prolongation was connected with syllable repetition in one study [21]. Repetition and prolongation were jointly examined in one study [25], and singularly, prolongation was explored in another [23].

### 3.3. Feature-Extraction Approach

Feature extraction stands as a pivotal step within speech-recognition systems, serving to convert raw audio signals into informative data for subsequent processing. It is a foundational element in the translation of spoken language into digital information, which facilitates human–technology communication. Previous studies have targeted various speech features, such as the Mel frequency cepstral coefficient (MFCC) [28]. Table 3 presents each type and its description.

In research, the method of feature extraction has undergone diverse exploration, with various techniques being employed to extract valuable insights from speech data. Among these methods, MFCC emerges as the most prevalent choice, with mentions in 6 out of the total 14 studies. The primary feature employed in automatic speech-recognition systems is the Mel frequency cepstral coefficient (MFCC). MFCC is obtained by applying the discrete cosine transform to the logarithm of the power spectrum, which is computed on a Mel scale frequency. It offers a more effective representation of speech, capitalizing on human auditory perception, and is widely applied in the majority of speech-recognition research. Its popularity can be attributed to its effectiveness in translating audio signals into a format that facilitates further analysis [29]. Table 3 also summarizes the computational methods to extract features from speech signals.

Nevertheless, in one particular study [21], a departure from the conventional MFCC approach is observed. Instead, the researchers opted for the utilization of the weighted MFCC (WMFCC). This distinctive choice stems from WMFCC’s unique ability to capture dynamic information inherent in speech samples, consequently bolstering the accuracy in detecting stuttering events. Furthermore, this alternative method offers the added advantage of reducing the computational overhead during the subsequent classification process, making it an intriguing avenue of exploration.

Spectrograms, a graphical representation of audio signals over time, have gained attention in multiple studies, notably in several studies [20,24,26]. These studies leverage spectrograms as a feature-extraction tool, emphasizing their utility in speech analysis.

Exploring more specialized domains, one study [17] delved into phonation features such as pitch, jitter, shimmer, amplitude perturbation quotient, pitch-period perturbation quotient, logarithmic energy, and the duration of voiceless speech. This nuanced approach offers a comprehensive understanding of the acoustic characteristics of speech.

Beyond the aforementioned methods, various other feature-extraction techniques have also been explored, including Ngram, character-based features, and utterance-based features. The combination of squeeze-and-excitation (SE) residual networks and bidirectional long short-term memory (BLSTM) layers, as witnessed in one study [14], illustrates the innovative strides taken to extract spectral features from input speech data, pushing the boundaries of feature extraction.

In Asci et al. [18], acoustic analysis of voice recordings was employed to further augment the array of feature-extraction methods applied. Intriguingly, Alharbi et al. [19] focused on word distance features, whereas Sheikh et al. [5] and Jouaiti and Dautenhahn [16] delved into the utilization of phoneme features. On a different note, Sheikh et al. [25] took a unique approach by extracting speaker embeddings from the ECAPA-time-delay neural network (TDNN) model and contextual embeddings from the Wav2Vec2.0 model, further enriching the feature-extraction landscape.

Lastly, Filipowicz and Kostek [27] introduced a pitch-determining feature into the signal processing toolkit, also exploring various 2D speech representations and their impact on classification results. This multifaceted exploration of feature-extraction techniques within the research realm highlights the dynamic and evolving nature of this crucial aspect of speech analysis.

### 3.4. Classifier Selection

In the world of ASD, different AI models have been employed in research with varying levels of accuracy and performance. These AI models have been investigated to see how well they can identify and understand stuttering, which has led to a range of results. Table 4 summarizes the computational methods for classifying speech features.

In our exploration of the existing literature, it was apparent that most studies have leaned toward applying deep learning models. By contrast, only 3 out of the 14 studies exclusively utilized machine learning. Additionally, several studies chose to combine both machine learning and deep learning models, whereas others opted for the creation of innovative architectural approaches.

Machine learning has emerged as a powerful tool in the domain of stuttering detection and classification. In this context, several studies have leveraged various traditional machine learning models, such as artificial neural network (ANN), K-nearest neighbor (KNN), and support vector machine (SVM), to develop faster diagnostic approaches.

Manjula et al. [8] employed ANN, which was fine-tuned using the adaptive fish swarm optimization (AFSO) algorithm. This ANN was purposefully trained to distinguish between various types of speech disfluencies, including repetitions, prolongations, and blocks, commonly observed in disfluent speech. The integration of the AFSO algorithm was instrumental in enhancing the network’s architectural design, thereby optimizing its performance in the disfluency classification task. In Sheikh et al. [25], a range of classifiers, including KNN, Gaussian back-end, and neural network classifiers, were applied for stuttering detection. Utilizing Wav2Vec2.0 contextual embedding-based stuttering-detection methods, the study achieved a notable improvement over baseline methods, showcasing superior performance across all disfluent categories. Asci et al. [18] utilized a support vector machine (SVM) classifier to extract acoustic features from audio recordings, achieving high accuracy in classifying individuals with stuttering. The study also identified age-related changes in acoustic features associated with stuttering, holding potential applications in clinical assessment and telehealth practice. In the ever-changing field of stuttering detection and classification, the use of deep learning techniques has led to significant advancements. The following studies highlighted how deep neural networks, especially the BLSTM and convolutional neural networks (CNNs), have played a transformative role in tackling the complexities of stuttering analysis.

In Kourkounakis et al. [20], a deep learning model that combines CNNs for extracting features from spectrograms with BLSTM layers for capturing temporal dependencies was introduced. This system outperformed existing methods in terms of detecting sound repetitions and revisions, boasting high accuracy and low miss rates across all stutter types. Moreover, Gupta et al. [21] employed the BLSTM model, achieving an impressive overall classification accuracy of 96.67% in detecting various types of stuttered events. This achievement was attributed to the utilization of WMFCC for feature extraction and BLSTM for classification, which outperformed conventional methods and displayed heightened accuracy in recognizing speech disfluencies. Furthermore, the utility of BLSTM emerged again in Jouaiti and Dautenhahn [23], where a deep neural network incorporating BLSTM was introduced for stuttering detection and dysfluency classification. The network’s architecture comprised multiple layers, including BLSTM layers, dense layers, batch normalization, and dropout layers, along with an embedding layer for processing phoneme-estimation data. Impressively, this network matched or exceeded state-of-the-art results for both stuttering detection and dysfluency classification.

Additionally, Al-Banna et al. [24] introduced a novel detection model comprising a 2D atrous convolutional network designed to learn spectral and temporal features from log Mel spectrogram data. This network architecture featured multiple layers, including convolutional layers with varying dilation rates, batch normalization, dropout layers, and softmax activation for predicting stuttering classes. When compared with other stuttering-detection methods, the proposed model exhibited superior performance, especially in the detection of prolongations class and fluent speech class. Meanwhile, in Prabhu and Seliya [26], a CNN-based classifier was designed for stutter detection, distinguishing itself from previous models relying on long short-term memory (LSTM)-based structures. This CNN-based model demonstrated high accuracy and precision, albeit with varying recall, making it exceptionally adept at achieving high F1 scores and surpassing other models across different datasets.

Several studies adopted a comprehensive approach by integrating both machine learning and deep learning models. These studies aim to identify the most effective approach for detecting and analyzing stuttering. In Alharbi et al. [19], a combination of conditional random fields (CRF) and BLSTM classifiers was utilized to detect and transcribe stuttering events in children’s speech. The study’s findings revealed that BLSTM outperformed CRFngram when evaluated using human-generated reference transcripts. Notably, the CRFaux variant, which incorporated additional features, achieved superior results compared to both CRFngram and BLSTM. Nevertheless, it is worth noting that when these classifiers were evaluated with ASR (automatic speech recognition) transcripts, all of them experienced a decrease in performance because of ASR errors and data mismatches.

In a parallel study, Sheikh et al. [25] used a range of classifiers, including KNN, Gaussian back-end, and neural network classifiers, to detect stuttering detection. Utilizing Wav2Vec2.0 contextual embedding-based stuttering-detection methods, the study obtained a noteworthy improvement over baseline methods, showcasing superior performance across all disfluent categories. Moreover, in Filipowicz and Kostek [27], various classifiers, including KNN, SVM, deep neural networks (ResNet18 and ResNetBiLstm), and Wav2Vec2, were evaluated for the classification of speech disorders. Notably, ResNet18 displayed superior performance over the other algorithms tested in the research.

Recently, in the field of ASD, various innovative approaches have surfaced to address the complex aspects of this speech condition. These studies embody significant advancements, each presenting fresh methods and structures for improving the comprehension of stuttering. Pravin and Palanivelan [17] presented a novel approach in the form of a deep long short-term memory (LSTM) autoencoder (DLAE) using long short-term memory (LSTM) cells, which are specialized recurrent neural network units designed for sequential data. The DLAE model was evaluated against various baseline models, including shallow LSTM autoencoder, deep autoencoder, and stacked denoising autoencoder, showing superior accuracy in predicting the severity class of phonological deviations. Meanwhile, Kourkounakis et al. [14] introduced FluentNet, a cutting-edge end-to-end deep neural network architecture designed exclusively for automated stuttering speech detection. FluentNet’s architecture comprises components such as SE-ResNet blocks, BLSTM networks, and an attention mechanism, achieving state-of-the-art results for stutter detection and classification across different stuttering types in both the *UCLASS* and *LibriStutter* datasets.

Furthermore, Sheikh et al. [22] proposed the StutterNet architecture, based on a time-delay neural network (TDNN) and specifically designed to detect and classify various types of stuttering. This architecture treats stuttering detection as a multiclass classification problem, featuring components such as an input layer, time-delay layers, statistical pooling, fully connected layers, and a softmax layer. Experimental results demonstrated the StutterNet model’s promising recognition performance across various stuttering types, even surpassing the performance of the ResNet + BiLSTM method in some cases, particularly in detecting fluent speech and core behaviors. Notably, the StutterNet model was also adopted by Sheikh et al. [6].

### 3.5. Preformance Evaluation

Within this section, an overview of the best accuracy across all studies is presented. In some of the studies, accuracy numbers were not explicitly provided, such as in [16], where the authors stated that the proposed AOANN effectively predicts the occurrences of repetitions, prolongations, and blocks with accuracy.

In contrast, the majority of the studies presented specific numerical data to illustrate their results. For instance, according to Al Harbi et al. [19], the BLSTM classifiers outperformed the CRF classifiers by a margin of 33.6%. However, incorporating auxiliary features for the CRFaux classifier led to performance enhancements of 45% compared to the CRF baseline (CRFngram) and 18% compared to the BLSTM outcomes.

Kourkounakis et al. [20] reported that their proposed model achieved a 26.97% lower miss rate on the *UCLASS* dataset compared to the previous state of the art. It also slightly outperforms the unidirectional LSTM baseline across all stutter types. Similarly, the DLAE model proposed by [17] outperformed the baseline models, achieving an AUC of 1.00 and a perfect test accuracy of 100%. This capability enables accurate discrimination between “mild” and “severe” cases of phonation deviation, ensuring precise assessment of speech disorders and avoiding any conflicting diagnoses.

The method of Gupta et al. [21] achieved the best accuracy of 96.67%, outperforming the LSTM model. Promising recognition accuracies were also observed for fluent speech (97.33%), prolongation (98.67%), syllable repetition (97.5%), word repetition (97.19%), and phrase repetition (97.67%). Furthermore, the FluentNet model proposed by Kourkounakis et al. [14] achieved an average miss rate and accuracy of 9.35% and 91.75% on the *UCLASS* dataset. The StutterNet model [22] outperformed the state-of-the-art method utilizing a residual neural network and BiLSTM, with a considerable gain of 4.69% in overall average accuracy and 3% in MCC. Also, the methodology proposed by Sheikh et al. [6] achieved a 4.48% improvement in F1 over the single-context-based MB StutterNet. Furthermore, data augmentation in the cross-corpora scenario improved the overall SD performance by 13.23% in F1 compared to clean training.

Moreover, the method proposed by Sheikh et al. [25] showed a 16.74% overall accuracy improvement over the baseline. Combining two embeddings and multiple layers of Wav2Vec2.0 further enhanced SD performance by up to 1% and 2.64%, respectively. During the training phase using *SEP-28K* + *FluencyBank* + *UCLASS* datasets, Jouaiti and Dautenhahn [23] achieved the following F1 scores: 82.9% for word repetition, 83.9% for sound repetition, 82.7% for interjection, and 83.8% for prolongation. In another training scenario using *FluencyBank* and *UCLASS*, the obtained F1 scores were 81.1% for word repetition, 87.1% for sound repetition, 86.6% for interjection, and 81.5% for prolongation.

The model proposed by Al-Banna et al. [24] surpassed the state-of-the-art models in detecting prolongations, with F1 scores of 52% and 44% on the *UCLASS* and *FluencyBank* datasets. It also achieved gains of 5% and 3% in classifying fluent speech on the *UCLASS* and *FluencyBank* datasets. In the study proposed by Prabhu and Seliya [26], interjection had the best performance with F1 score: 97.8%. Furthermore, in [18], machine learning accurately differentiated individuals who stutter from controls with an 88% accuracy. Age-related effects on stuttering were demonstrated with a 92% accuracy when classifying children and younger adults with stuttering. Additionally, in [27], ResNet18 was able to classify speech disorders at the F1 measure of 93% for the general class.

## 4. Discussion

**Figure 3:**
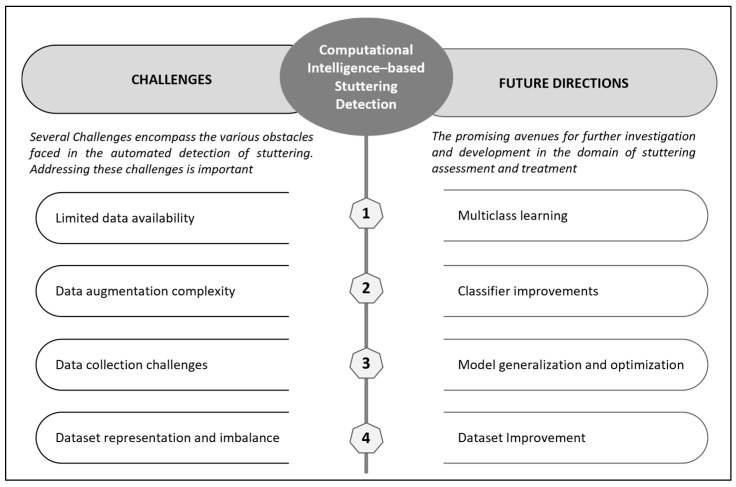
Challenges and future directions of computational intelligence-based stuttering detection.

In this section, the importance of understanding the challenges and identifying future directions in computational intelligence-based stuttering detection is examined further. By exploring these aspects, we gain valuable insights into the current limitations of existing approaches and pave the way for advancements in the field. Figure 3 provides insights into the challenges and future directions of computational intelligence-based stuttering detection.

### 4.1. Challenges

In this section, an overview of the challenges that automatic stuttering-identification systems encounter is provided, and possible solutions that could be explored in the field of stuttering research are suggested. Although there have been notable developments in the automated detection of stuttering, several issues must still be addressed to ensure a robust and effective identification of stuttering.

A significant obstacle that should be addressed is the limited availability of data for research in stuttering identification. A notable challenge is the scarcity of natural speech datasets that include disfluent speech. The limitations posed by the availability of a limited dataset have been a recurring concern in several studies [19,20,23,25]. This constraint can significantly impact the outcomes of their proposed methods, which often leads to results that do not meet expectations. The deficiency in extensive and diverse datasets has emerged as a pivotal factor that influences the overall performance and robustness of the methods explored in these studies. Hence, addressing the challenge of dataset scarcity is a fundamental step toward enhancing the accuracy and reliability of research findings in this domain.

Medical data collection is generally a costly and resource-intensive endeavor, and stuttering research is no exception in this regard [25]. Moreover, the complexity of the stuttering domain is compounded by the need for a diverse set of speakers and sentences for comprehensive analysis. One of the obstacles that contribute to the scarcity of available datasets is the challenge of data collection itself. This challenge arises since it involves organizing meetings and recording sessions with PWS while they engage in spontaneous speech. This approach is necessary to capture authentic instances of stuttering speech considering that requesting PWS to read from a predetermined list can often result in a reduction in the frequency of stuttering occurrences [30].

Sheikh et al. [6] introduced a possible solution to the challenge posed by the limited availability of datasets in the field. They proposed that data augmentation could prove beneficial in the context of stuttering research. However, notably, the application of data augmentation in the realm of stuttering is not a straightforward process. This complexity arises because several conventional data augmentation techniques, such as time stretch and the fundamentally high rate of speech, can significantly alter the underlying structure of stuttering speech samples.

To make data augmentation more effective for stuttering research, specialized data augmentation techniques tailored specifically to the unique characteristics of stuttering speech must be developed. Such domain-specific data augmentation methods would enable researchers to enhance their datasets while preserving the vital features of disfluent speech, ultimately contributing to more accurate and meaningful results in this field of study.

Another significant concern that affects the outcomes of proposed procedures in various studies is the class imbalance within the available datasets, as highlighted by Jouaiti and Dautenhahn [23] and Filipowicz and Kostek [27]. This problem arises from several factors, one of which is the limited availability of datasets. Notably, certain datasets may exhibit an underrepresentation of specific types of core stuttering behaviors, such as the case of “prolongations” in the *FluencyBank* dataset. Stuttering, which is a highly diverse speech disorder, can manifest in diverse ways among individuals. Some individuals may predominantly display repetitions, whereas others may experience more prolonged speech sounds or blocks. This inherent variability in stuttering presentations contributes to the disparities observed in stuttering datasets.

Speech data collection from PWS, especially in authentic conversational settings, introduces its own set of challenges. Some core stuttering behaviors may occur less frequently or may be less perceptible because PWS may be more likely to stutter when they are feeling anxious or stressed [30]. Moreover, PWS may be more likely to use avoidance strategies (e.g., pausing and substituting a word) in authentic conversational settings, such as avoiding words or phrases that they know are likely to trigger stuttering, rendering it more challenging to capture the events during data collection endeavors [31]. Furthermore, stuttering datasets often suffer from limited size because of the relatively low prevalence of stuttering in the general population. This limited sample size increases the likelihood of imbalances that arise purely by chance. Fundamentally, the issue of class imbalance in stuttering datasets stems from multifaceted factors, which include the diverse nature of stuttering, data collection challenges, and the inherent constraints associated with dataset size. Recognizing and addressing these factors are necessary steps in striving for more balanced and representative datasets in stuttering research.

### 4.2. Future Directions

Based on the findings of our systematic review, we have identified several potential future directions for research in the field. These directions include the exploration of multiclass learning techniques, improvements in classifier algorithms, advancements in model generalization and optimization methods, as well as enhancements in dataset quality and diversity. These areas present promising avenues for further investigation and development in the domain of stuttering assessment and treatment.

#### 4.2.1. Multiclass Learning

Numerous researchers have sought to enhance their systems by incorporating the concept of multiclass learning. This means that instead of restricting their proposed models to identifying only one type of stuttering at a time, these models can recognize multiple stuttering types concurrently. PWS can exhibit various forms of stuttering within a single sentence, which occur simultaneously in their speech.

Kourkounakis et al. [20] aimed to build upon existing models and embarked on research regarding multiclass learning for different stuttering types. Considering that multiple stuttering types can manifest simultaneously in a sentence (e.g., “I went to uh to to uh to”), this approach has the potential to yield a more robust classification of stuttering.

Furthermore, Sheikh et al. [22] outlined future work in which they would examine thoroughly the realm of multiple disfluencies. They intended to explore advanced variations of TDNN for stuttering detection in real-world settings. This advancement aims to address the complexity of stuttering, where different disfluency types can co-occur, contributing to a more comprehensive understanding of stuttering patterns in spontaneous speech.

#### 4.2.2. Classifier Improvements

This research strongly highlights the importance of enhancing the classifiers as a central focus for future work. Improving the classifiers is a pivotal aspect of our future research agenda, which reflects its importance in achieving more accurate and effective ASR classification. 

Alharbi et al. [19] proposed enhancements to the ASR stage, intending to reduce the word error rate. They also suggested exploring alternative methods for detecting prolongation events, which proved challenging using the current approach.

Pravin and Palanivelan [17] aimed to employ deep learning for the solitary classification of disfluencies, contributing to a more descriptive severity assessment for subjects and enhanced self-assessment. Nevertheless, notably, an increase in the number of training epochs resulted in longer model run times, which requires consideration for future improvements. Moreover, Gupta et al. [21] suggested the exploration of different feature-extraction and classification techniques to improve stuttering detection. Kourkounakis et al. [14] proposed experimenting with FluentNet’s architecture, potentially implementing different attention mechanisms, including transformers, to investigate their impact on results.

Sheikh et al. [6] proposed exploring the combination of various types of neural networks in stuttering detection to pinpoint precisely where stuttering occurs in speech frames. Furthermore, investigating different context variations, depths, and convolutional kernel numbers in stutter detection poses a promising area of focus. The study also identified blocks as particularly difficult to detect, prompting further analysis and ablation studies on speakers with hard-to-determine disfluencies.

Future work involves exploring self-supervised models that utilize unlabeled audio data, building upon the research of Sheikh et al. [25], which aimed at fine-tuning the Wav2Vec2.0 model to identify and locate stuttering in speech frames. Al-Banna et al. [24] suggested the incorporation of atrous spatial pyramid pooling and local and global attention mechanisms to enhance detection scores. 

Prabhu and Seliya [26] identified potential enhancements to the model as possibly including the incorporation of LSTM layers to capture temporal relationships in data and improve performance. The possibility of utilizing different machine models, such as LSTM models, has also been considered for better data interpretation with the *SEP-28k* dataset. Finally, Filipowicz and Kostek [27] proposed extending the training duration for each model and potentially expanding the ResNet18 model with additional convolutional layers, which are dependent on available resources.

#### 4.2.3. Model Generalization and Optimization

Several researchers have stressed the importance of a crucial area for future research: enhancing their models’ ability to generalize. This means making their models better at working effectively in different situations, not just the specific ones for which they were originally designed. The reason behind this recommendation is slightly straightforward: Researchers want their models to be versatile and adaptable. They understand that a model’s success should not be confined to specific datasets or conditions. By focusing on improving generalization, researchers aim to strengthen their methods, which enables them to handle a wide range of real-world challenges and variations effectively.

Kourkounakis et al. [14], for example, proposed the idea of conducting more studies that bridge synthetic datasets such as *LibriStutter* with real-world stutter datasets. They suggested exploring domain adaptation techniques, which include the use of adversarial networks, to enhance the transfer of learning and create more adaptable and broadly applicable solutions. Furthermore, researchers could investigate optimizing various parameters concurrently, such as context, filter bank size, and layer dimensions within the proposed system.

Additionally, Sheikh et al. [25] suggested that it would be interesting to study how well the proposed method can generalize across multiple datasets. This exploration would shed light on the method’s adaptability and effectiveness in various data scenarios. Finally, Al-Banna et al. [24] recommended investigating ways to improve model generalization and robustness by analyzing different datasets and using domain-adaptation techniques. These recommendations collectively highlight researchers’ dedication to advancing methods that are not only effective in specific situations but also excel in various real-world contexts.

#### 4.2.4. Dataset Improvement

In terms of dataset enhancement, Prabhu and Seliya [26] highlighted the potential for improving the *SEP-28k* dataset. Notably, the *SEP-28k* dataset is recognized for its robustness, comprising a substantial volume of stuttering data that serve as a valuable resource for model development. Nevertheless, this dataset still offers room for refinement and optimization.

A key proposal involves revising the labeling scheme currently applied to each 3-second audio clip. By creating more specific labels with distinct start and end points, the dataset could pave the way for the development of more effective classifiers. Such an adjustment would render disfluent and fluent events entirely independent of each other, potentially facilitating the detection of these events within arbitrary speech contexts.

## 5. Conclusions

Stuttering is a complex speech disorder that necessitates accurate detection for effective assessment and treatment. This paper has discussed the challenges, advancements, and future directions in computational intelligence-based stuttering detection.

The challenges of limited datasets and dataset imbalance were determined, with proposed solutions including specialized data-augmentation techniques and balanced dataset creation. Advancements in stuttering detection using computational intelligence techniques have shown promising results from employing various algorithms and feature-extraction methods.

Future research directions include multiclass learning approaches, classifier enhancements, model generalization, and optimization. When these challenges and the exploration of future directions are addressed, we can enhance the accuracy and reliability of stuttering-detection systems, benefiting individuals who stutter and improving their quality of life.

## Figures and Tables

**Table 1:** Benchmark datasets.

Dataset	Classes	Description
*UCLASS* (2009) [11]	Interjection, sound repetition, part-word repetition, word repetition, phrase repetition, prolongation, and no stutter	The *University College London’s Archive of Stuttered Speech* (*UCLASS*) is a widely used dataset in stuttering research. It includes monologs, conversations, and readings, totaling 457 audio recordings. Although small, UCLASS is offered in two releases by UCL’s Department of Psychology and Language Sciences. Notably, *UCLASS3* release 1 contains 138 monolog samples, namely 120 and 18 from male and female participants, respectively, from 81 individuals who stutter, aged 5–47 years. Conversely, release 2 contains a total of 318 monologs, reading, and conversation samples from 160 speakers suffering from stuttering, aged 5–20 years, with samples from 279 male and 39 female participants. Transcriptions, including orthographic versions, are available for some recordings, making them suitable for stutter labeling.
*VoxCeleb* (2017) [15]	The dataset does not have classes in the traditional sense, as it is more focused on identifying and verifying individual speakers	It is developed by the VGG, Department of Engineering Science, University of Oxford, UK. It is a large-scale dataset designed for speaker-recognition and verification tasks. It contains a vast collection of speech segments extracted from celebrity interviews, talk shows, and online videos. This dataset covers a diverse set of speakers and is widely employed in research that is related to speaker recognition, speaker diarization, and voice biometrics.
*SEP-28k* (2021) [12]	Prolongations, repetitions, blocks, interjections, and instances of fluent speech	Comprising a total of 28,177 samples, the *SEP-28k* dataset stands as the first publicly available annotated dataset to include stuttering labels. These labels encompass various disfluencies, such as prolongations, repetitions, blocks, interjections, and instances of fluent speech without disfluencies. Alongside these, the dataset covers nondisfluent labels such as natural pauses, unintelligible speech, uncertain segments, periods of no speech, poor audio quality, and even musical content.
*FluencyBank* (2021) [13]	Individuals who stutter (IWS) and individuals who do not stutter (IWN)	The *FluencyBank* dataset is a collection of audio recordings of people who stutter. It was created by researchers from the United States and Canada and contains over 1000 h of recordings from 300 speakers. The dataset is divided into two parts, namely research and teaching. The research data are password-protected, and the teaching data are open-access. The teaching data include audio recordings of 10 speakers who stutter, transcripts, and annotations of stuttering disfluencies. The dataset is valuable for researchers and clinicians studying stuttering.
*LibriStutter* (2021) [14]	Sound, word, and phrase repetitions; prolongations; and interjections.	The *LibriStutter* dataset is a corpus of audio recordings of speech with synthesized stutters. It was created by the Speech and Language Processing group at Queen’s University in Canada. The dataset contains 100 h of audio recordings of 10 speakers, each of whom stutters differently. The stutters were synthesized via a technique known as the hidden Markov model. It is a valuable resource for researchers who are developing automatic speech-recognition (ASR) systems for people who stutter. The dataset can also be used to train models for detecting and classifying different types of stutters.

**Table 2:** Classification of stuttering type.

Type	Definition	Example
Repetition	Repeating a sound, syllable, or word multiple times.	“I-I-I want to go to the park.”
Prolongation	Extending or elongating sounds or syllables within words.	“Sssssend me that email, please.”
Block	Temporary interruption or cessation of speech flow.	“I can’t... go to the... park tonight.”
Interjection	Spontaneous and abrupt interruption in speech with short exclamations.	“Um, I don’t know the answer.”
Sound repetitions	Repeating individual sounds within a word.	“Th-th-that movie was great.”
Part-word repetitions	Repetition of part of a word, usually a syllable or sound.	“Can-c-c-come over later?”
Word repetitions	Repeating entire words within a sentence.	“I like pizza, pizza, pizza.”
Phrase repetitions	Repeating phrases or groups of words.	“He said, “he said it too.”
Syllable repetition	Repeating a syllable within a word.	“But-b-but I want to go.”
Revision	Rewording or revising a sentence during speech to avoid stuttering.	“I’ll take the, um, the bus.”

**Table 3:** Computational methods for the feature-extraction phase.

Method	No. of Studies	Ref.
Mel frequency cepstral coefficient (MFCC)	6	Sheikh et al., 2023 [6] Manjula et al., 2019 [16] Sheikh et al., 2021 [22] Jouaiti and Dautenhahn, 2022 [23] Sheikh et al., 2022 [25] Filipowicz and Kostek, 2023 [27]
Weighted MFCC (WMFCC)	1	Gupta et al., 2020 [21]
Spectrograms	3	Kourkounakis et al., 2020 [20] Al-Banna et al., 2022 [24] Prabhu and Seliya, 2022 [26]
Phonation features	1	Pravin and Palanivelan, 2021 [17]
Ngram	1	Alharbi et al., 2020 [19]
Character-based features	1	Alharbi et al., 2020 [19]
Utterance-based features	1	Alharbi et al., 2020 [19]
Acoustic analysis of voice recordings	1	Asci et al., 2023 [18]
Word distance features	1	Alharbi et al., 2020 [19]
Phoneme features	2	Sheikh et al., 2023 [6] Sheikh et al., 2022 [25]
Squeeze-and-excitation (SE) residual networks	1	Kourkounakis et al., 2021 [14]
Bidirectional long short-term memory (BLSTM) layers	1	Kourkounakis et al., 2021 [14]
Speaker embeddings from the ECAPA-TDNN model	1	Sheikh et al., 2022 [25]
Contextual embeddings from the Wav2Vec2.0 model	1	Sheikh et al., 2022 [25]
Pitch-determining feature	1	Filipowicz and Kostek, 2023 [27]
Two-dimensional speech representations	1	Filipowicz and Kostek, 2023 [27]

**Table 4:** Computational methods for classifying speech features.

Method	Ref.
Artificial neural network (ANN)	Manjula et al., 2019 [16] Sheikh et al., 2022 [25]
K-nearest neighbor (KNN)	Sheikh et al., 2022 [25] Filipowicz and Kostek, 2023 [27]
Gaussian back-end	Sheikh et al., 2022 [25]
Support vector machine (SVM)	Asci et al., 2023 [18] Filipowicz and Kostek, 2023 [27]
Bidirectional long short-term memory (BLSTM)	Pravin and Palanivelan, 2021 [17] Asci et al., 2023 [18] Alharbi et al., 2020 [19] Gupta et al., 2020 [21]
Convolutional neural networks (CNNs)	Kourkounakis et al., 2020 [20] Prabhu et al., 2022 [26]
Two-dimensional atrous convolutional network	Al-Banna et al., 2022 [24]
Conditional random fields (CRF)	Alharbi et al., 2020 [19]
ResNet18	Filipowicz and Kostek, 2023 [27]
ResNetBiLstm	Filipowicz and Kostek, 2023 [27]
Wav2Vec2	Filipowicz and Kostek, 2023 [27]
Deep LSTM autoencoder (DLAE)	Pravin and Palanivelan, 2021 [17]
FluentNet	Kourkounakis et al., 2021 [14]
StutterNet	Sheikh et al., 2023 [6] Sheikh et al., 2021 [22]
